# Diagnostic precision of image-guided multisampling core needle biopsy of suspected lymphomas in a primary care hospital

**DOI:** 10.1038/sj.bjc.6605059

**Published:** 2009-04-28

**Authors:** P Loubeyre, T A McKee, M Copercini, A Rosset, P-Y Dietrich

**Affiliations:** 1 Division of Radiology, Department of Imaging and Medical Informatics, Hôpitaux Universitaires de Genève. Geneva University Hospitals, Suisse, Switzerland; 2 Division of Clinical Pathology, Department of Medical Genetics and Laboratory Medicine, Hôpitaux Universitaires de Genève. Geneva University Hospitals, Suisse, Switzerland; 3 Division of Oncology Service, Department of Internal Medicine, Hôpitaux Universitaires de Genève. Geneva University Hospitals, Suisse, Switzerland

**Keywords:** core needle biopsy, lymphoma, molecular analysis

## Abstract

We evaluated the diagnostic quality of image-guided multisampling core needle biopsy (CNB) in patients investigated for suspected lymphoma in a primary care hospital. A total of 112 patients were consecutively assessed during a 3-year period. There were 80 lymphoid site biopsies and 32 non-lymphoid site biopsies. Eight to nine cores were obtained from different parts of the biopsy site. Two cores were systematically frozen, allowing for further morphological, immunochemistry and molecular studies. The diagnostic yield of CNB for malignancy was 100%. Only 47% (41/87) of patients with initial suspicion of lymphoma were finally diagnosed with Lymphoma. The diagnostic yield of CNB for lymphoma typing was 98% (62/63), according to the WHO classification. The diagnostic yield of CNB for complete lymphoma subtyping/grading was 86% (54/63). The diagnostic yield of CNB for a definite diagnosis of benignity was only 47% (8/17). In a primary care setting, multisampling CNB is a minimally invasive, and very accurate procedure for confirming malignancy in patients with suspected lymphoma, presenting with superficial/deep-seated, lymphoid/non-lymphoid site targets. With a very high diagnostic yield for lymphoma typing and a high diagnostic yield for complete lymphoma subtyping/grading a therapeutic decision can be taken in most patients.

There is a clear trend in oncological diagnostic work-up, which was initiated in breast imaging, towards performing multisampling core needle biopsy (CNB) of suspected lesions to obtain samples of good quality and quantity. However, the excisional biopsy of enlarged lymph nodes is still advocated as the gold standard in the diagnostic evaluation of lymphoma (Dreyling, 2008; Engert and Dreyling, 2008; Tilly and Dreyling, 2008). Nonetheless, in several centres, percutaneous image-guided CNB, which is a minimally invasive procedure, is used as an alternative to excisional biopsy for diagnosing lymphomas (de Kerviler *et al*, 2000; Demharter *et al*, 2001; Screaton *et al*, 2002; Balestreri *et al*, 2005; Li *et al*, 2005; Sklair-Levy *et al*, 2005; Lachar *et al*, 2007; de Kerviler *et al*, 2007; de Larrinoa *et al*, 2007). This is particularly true if suspected nodes or masses are deep seated or if the clinical condition of patients is severely impaired. This procedure needs further validation, particularly relating to its use in a primary care hospital, where a large differential diagnosis for peripheral/deep-seated lymph node(s) or masse(s) exists. In our institution, image-guided large-cutting coaxial multisampling CNB is the routine diagnostic approach of all suspected peripheral/deep-seated lymph nodes or tissular masses. We retrospectively evaluated the diagnostic quality of this procedure during a 3-year period, in a series of consecutive patients investigated for suspected lymphoma.

## Materials and methods

### Patients

All patients of the Internal Medicine Department with a presumptive diagnosis of lymphoma on the basis of clinical presentation and imaging findings were referred to the Imaging Department for image-guided CNB. Before the CNB procedure, a review of all imaging and medical records was carried out with the medical staff to choose the most appropriate biopsy target. No patient with suspected lymphoma had fine-needle aspiration cytology before CNB. Informed consent for the procedure was obtained from each patient.

Clinical staging of presumptive LPD was based on distribution of disease according to the Ann Arbor clinical staging classification.

We retrospectively evaluated CNB procedures in all patients (47 women, 65 men; age range, 20–91 years; mean age, 58 years) assessed by image-guided CNB for suspected LPD, during a 3-year period. No patient was excluded because of insufficient material on CNB. CNB procedure was evaluated in 112 patients.

Eighty-seven patients had an initial presentation of suspected lymphoma. Of these, 11 had a previous malignancy (urothelial, 4; breast, 1; colic, 1; pulmonary, 1; melanoma, 2; ovary, 1; uterus, 1) but clinical presentation, localisation and delay of appearance of suspected lesions were rather suggestive of lymphoma.

Twenty-five patients had a previous history of lymphoma. Indication for biopsy was a suspicion of relapsed or histological transformation of the disease.

Biopsy procedures were performed on an outpatient basis (70%), unless the patient status necessitated hospitalisation (30%).

### Biopsy sites and image-guided biopsy procedure

All patients had image-based staging with cervico-thoraco-abdominal-enhanced CT. When several lymphoid/non-lymphoid site targets were available, biopsy of peripheral targets was preferred over biopsy of deep-seated targets when both were present. There were 80 lymphoid site biopsies (peripheral, 37; deep seated, 43) and 32 non-lymphoid site biopsies (peripheral, 6; deep seated, 26; Table 1). Peripheral sites were biopsied under sonographic guidance whereas deep-seated sites were biopsied under CT guidance.

A coagulation screen was only obtained before a deep-seated target biopsy procedure (exclusion criteria for the biopsy were a prothrombin time <60% or a platelet count <50 000 per *μ*l). Premedication was generally not given. The biopsy procedure was standardised and performed by experienced radiologists (PL or MC).

In the case of biopsy under contrast-enhanced CT guidance, selected images were obtained in the area of interest (1.5 mm beam collimation, 3 mm reconstruction thickness, 3 mm reconstruction interval, 1.25 pitch, 0.75 s rotation time, 120 Kvp and 180 MAs). Patients were told to breathe in a slow, regular and shallow manner throughout the biopsy procedure and during acquisition of images. It is our personal experience that breathing instructions, such as suspended inspiration or expiration, raise the difficulty of the procedure, because they are not always reproducible. Local anaesthesia using 5–10 ml of xylocaine was carefully performed along the chosen tract of the biopsy procedure, and then an adjustable coaxial 17-gauge automated CNB system (Temno Biopsy System; Allegiance Healthcare Corporation, McGaw Park, IL, USA) was used. The system consists of a 17-gauge (1.48 mm diameter) outer cannula with an introducer stylet that is used for positioning the outer cannula. Once its tip is on the surface of the lesion, the stylet is taken out and an 18-gauge (1.25 mm diameter) central cutting needle biopsy is passed through the cannula for biopsy sample. The sample length can be selected to a maximum of 24 mm by turning a coaxial bolt. Eight/nine cores, considered macroscopically adequate by the radiologist, were obtained from different parts of the lesion by slightly modifying the orientation of the cannula. The criterion for a macroscopically adequate core was extraction of a sharply cut tissue core. In the case of large lesions, biopsy of the peripheral portion of the lesion was preferred to avoid possible biopsy of necrotic tissue in the central region. In lymph nodes, the peripheral capsule was systematically sampled. Duration of sample harvesting was less than 15 min. Biopsy samples were separated and immediately sent to the laboratory in part unfixed and in part fixed (in buffered formaldehyde) for immediate processing. A ratio of 2/3 fixed and 1/3 unfixed was generally used (three cores were unfixed). This material was forwarded along with a sheet that detailed the clinical history of the patient.

### Search for complications

After removal of the biopsy system, immediate postbiopsy complications were monitored. In cases of a deep-seated target biopsy procedure, outpatients were transferred to a holding unit and kept under observation for 2 h and were discharged if there were no significant complications. They were encouraged to contact their physicians if they developed symptoms after leaving hospital.

### Histopathological examination

A haemato-histopathologist (TMK) reviewed all specimens. The priority with fresh (unfixed) samples was given to storage in a tissue bank to allow subsequent molecular analysis if judged necessary (two samples). Remaining unfixed material was dissociated and analysed by flow cytometry.

Fixed samples were embedded in paraffin and stained with haematoxylin and eosin and Giemsa stains. The histological appearance dictated the choice of antibodies for immunohistochemical stains. Lymphoid infiltrates were characterised using an appropriate selection of antibodies (from CD45, CD3, CD4, CD5, CD8, CD10, CD15, CD 20, CD21, CD23, CD30, CD43, CD79a, BCL-1, BCL-2, BCL-6, IRF4 and MIB1). If necessary, sections were cut for analysis by fluorescence *in situ* hybridisation using selected probes (from IGH, CCND1, BCL2, MALT, MYC; 4 cases). Fresh frozen material was available to allow T- or B-cell clonality studies and the translocations [1] (14;18) and (11;14) to be characterised using primer sets defined by the BIOMED consortium (29 cases), and also for further molecular analysis if necessary (identification of microorganisms such as mycobacteria, EBV, HHV-8, etc.; 7 cases). Flow cytometry was performed in further seven cases.

The recently established revised European–American classification of lymphoid neoplasmas and the subsequently adopted and updated World Health Organisation (WHO) classification of tumours of haematopoietic and lymphoid tissues were used to classify lymphomas.

Metastatic tumours were identified by standard histological analysis and immunochemistry if necessary. Histopathology reports were reviewed for evaluation of diagnostic yield of CNB.

### Methodology

To estimate the validity of CNB in the context of suspected LPD seen in a primary care institution, the following parameters were analysed:

The diagnostic yield of CNB to establish the diagnosis of a malignant disease (lymphoma or others) is the ratio of the number of malignant diagnoses on CNB divided by the number of malignant diagnoses at final diagnostic work-up.

The diagnostic yield of CNB to establish the diagnosis of a benign process is the ratio of the number of definite benign diagnoses made by CNB by the number of benign diagnoses at final diagnostic work-up (i.e., subsequent surgical biopsy, laboratory tests or clinical follow-up during a 1-year period).

The diagnostic yield of CNB for lymphoma typing is the ratio of the number of cases for which a precise diagnosis of lymphoma was made on CNB, divided by the number of the entire cohort of lymphomas.

The diagnostic yield of CNB for lymphoma subtyping is the ratio of the number of cases for which the subtype (e.g., germinal centre *vs* activated type for DLBCL) or the grading (e.g., for follicular lymphomas (FLs)) was made on CNB, divided by the number of the entire cohort of lymphomas.

For cases of incomplete lymphoma typing/subtyping/grading, retrieved data were used to assess whether subsequent surgical biopsy was proposed or CNB results were considered as sufficient for adapted therapeutic decision.

## Results

Of the 112 patients addressed for CNB, 87 had an initial presentation of suspected lymphoma and 25 had suspected relapsed/transformed lymphoma.

The tolerance of the procedure was excellent. No patient had severe pain requiring the interruption of the biopsy. On clinical or imaging follow-up, only minor complications were noted, one small pneumothorax, a 3-cm diameter cervical haematoma and a 4 cm diameter adrenal haematoma. They all resolved spontaneously.

Histopathological analysis of CNB samples showed malignant diagnoses in 95 patients (63 lymphomas and 32 non-lymphomas), benign diagnoses in 8 patients and non-malignant diagnoses without definitively excluding lymphoma in 9 patients (Tables 2). The diagnostic yield of CNB for malignancy (number of malignant diagnoses on CNB/number of malignant diagnoses at final diagnostic work-up) was 100%, indicating that no case of malignancy was missed by a careful CNB examination. It is noteworthy that only 45% (41/87) of patients with initial suspicion of lymphoma were finally diagnosed with lymphoma, with CNB revealing 32 cases of other cancers (metastatic or primary) and 14 cases of non-malignant processes. These data strengthen the potential of the CNB approach in the context of a primary care hospital where a large diversity of diagnoses is expected. It is also noteworthy that 10 out of 25 patients with suspected relapsed/transformed lymphoma had a CNB diagnosis different from initial lymphoma diagnosis (Table 3).

In contrast, the diagnostic yield of CNB for a definite diagnosis of benignity was only 47% (8/17). This underlines the difficulty of obtaining a precise diagnosis of a benign disorder with CNB samples. However, except for one patient who was lost to follow-up, the absence of lymphoma was confirmed in all other eight cases. In two cases of necrotising granulomatous inflammation, the diagnosis of tuberculosis was established by cultures; two patients with peripheral lymph nodes spontaneously recovered within a few months and four patients underwent surgical exploration to excise entire lymph nodes, which confirmed the benign nature of the disease (Table 4). The most important endpoint of this study was to define whether CNB is an appropriate strategy for the diagnosis of lymphoma arising in a patient population from a primary care institution. The diagnostic yield of CNB for lymphoma typing was 98% (62/63; Table 2). For one patient only, the material provided by CNB was insufficient to make the differential diagnosis between Hodgkin's lymphoma and anaplastic large cell lymphoma. He declined surgical exploration and was lost to follow-up. For all other patients, a precise diagnosis according to the WHO classification was established.

A detailed description of nodal architecture, cell characteristics and interstitial fibrosis may influence the treatment choice or intensity of certain lymphoma entities. The diagnostic yield of CNB for complete lymphoma subtyping/grading was 86% (54/63). Among the eight cases with incomplete lymphoma subtyping/grading, there were four classical Hodgkin's lymphoma for which the histological subtype could not be determined. However, the treatment decision for early or advanced stages of Hodgkin's lymphoma relies on well-defined prognostic factors that do not include the subtype. There were two DLBCL, for whom there was not enough material to discriminate between germinal centres and activated subtypes, but it has not yet been agreed that treatment intensity should consider these parameters. There were two patients with a history of low-grade lymphoma. The first case was a patient relapsing five years after the diagnosis of grade 1 FL. The biopsy of a retroperitoneal lymph node confirmed the diagnosis of FL, but a precise grading was not possible. However, the patient was symptomatic with important tumour masses and his treatment was dictated by these clinical parameters. The last case was a patient with a known low-grade lymphoma, which was unclassifiable based on in-depth analysis of an entire lymph node. At the time of relapse, 3 years later, the same pathological characteristics were found in CNB samples, including the same B-cell receptor rearrangement. Due to the indolent clinical course, a wait and see attitude was preferred.

We wondered whether technical CNB difficulties related to the size or the location of lymph nodes/masses could explain incomplete lymphoma typing/subtyping/grading. For the nine cases of incomplete lymphoma typing/subtyping/grading (one case of incomplete lymphoma typing and eight cases of incomplete lymphoma subtyping/grading), diameters of biopsy targets were random, ranging from 15 to 90 mm (mean, 37.8 mm). In contrast, biopsy sites were deep seated in seven out of nine cases suggesting that the location of the target, rather than the size, could constrain an optimal sampling technique.

## Discussion

A precise histological diagnosis using current classification systems is a critical step for guiding appropriate treatment choices in lymphoma. This requires sufficient material to allow recognition of complex architectural patterns, realisation of numerous immunophenotyping procedures and, more and more frequently, complex molecular analyses that are often the final proof for a suspected diagnosis. Consequently, entire lymph node resection is still considered as the method of choice, mainly for peripheral lymph nodes. However, deep-seated lymph nodes or masses remain a challenge. In this situation, surgery may be difficult to achieve within a few days (a short period which is often necessary in the context of aggressive lymphoma), is expensive and may induce several postoperative complications that further delay the initiation of curative chemotherapy.

Needle aspiration cytology (NAC) has been seen as a valuable alternative diagnostic procedure. However, it requires specific cytopathological expertise (not available in all centres, at least in Europe), and has important limitations (e.g., there is no information about nodal architecture, less material for immunohistochemical and molecular analysis,…). Such intrinsic drawbacks are more and more critical with the current view of lymphoma diagnosis that requires the coordinated interpretation of structural, phenotypic and genotypic characteristics. Some authors reported a high diagnostic rate was with NAC (Goldschmidt *et al*, 2003; Agid *et al*, 2005), and others reported that NAC misguided lymphoma diagnosis (Hehn *et al*, 2004). Nonetheless, it is clear that cytological analysis can provide a rapid diagnosis in specific clinical situations such as (1) metastatic carcinomas or (2) aggressive lymphoma, where a rapid diagnosis allows therapy to be initiated without the delay that results from fixation and processing of biopsy material (touch prints of biopsies can also be used for this purpose).

Image-guided CNB is a minimally invasive procedure offering a distinct advantage over FNAC in that it obtains intact tissue. A strategy for obtaining a large amount of tissue is the use of an automated large-cutting biopsy gun with a coaxial technique. The main advantage of the coaxial technique is its ability to sample several core specimens with a single biopsy tract. A cannula is first inserted through the skin and towards the lesion. The cannula remains in position during the sampling procedure, thus decreasing the potential risk of postprocedure complications and tumour cell spreading. By slightly modifying the orientation of the cannula, multiple samples can be obtained in different representative portions of the lesion, providing valuable information (while partial) on nodal architecture in most cases. Thus, image-guided large-cutting CNB has been proposed as the initial procedure for the diagnosis of deep-seated lymphomas (de Kerviler *et al*, 2000; Demharter *et al*, 2001; Balestreri *et al*, 2005; Li *et al*, 2005; Sklair-Levy *et al*, 2005; Lachar *et al*, 2007; de Larrinoa *et al*, 2007), and in some institutions for suspect peripheral lymph nodes (Screaton *et al*, 2002; de Kerviler *et al*, 2007). Although variable endpoints were analysed in those studies with highly diverse designs, CNB appears to be a valuable approach with sensitivity for diagnosing lymphoma at 87–89% (Demharter *et al*, 2001; Balestreri *et al*, 2005), overall diagnostic yield at 84% (Sklair-Levy *et al*, 2005), unequivocal diagnosis of lymphoma at 91% (Lachar *et al*, 2007), overall diagnostic accuracy for lymphoma typing at 88% (de Larrinoa *et al*, 2007), sufficient information such that a therapeutic decision could be made at 96% (de Kerviler *et al*, 2007) and diagnosis of lymphoma with subtyping ranging from 76 to 85% (de Kerviler *et al*, 2000; Li *et al*, 2005).

However, the validity of CNB for lymphoma diagnosis is still controversial, and entire lymph node removal is often proposed as the best procedure (Dreyling, 2008; Engert and Dreyling, 2008; Tilly and Dreyling, 2008). Moreover, most of the studies (de Kerviler *et al*, 2000; Demharter *et al*, 2001; Screaton *et al*, 2002; Balestreri *et al*, 2005; Li *et al*, 2005; Sklair-Levy *et al*, 2005; de Kerviler *et al*, 2007; de Larrinoa *et al*, 2007; Lachar *et al*, 2007) include patients with a high probability of lymphoma diagnosis assessed in reference centres for lymphoproliferative diseases. This study was designed to determine whether CNB can also be recommended in the context of a primary care hospital where a large range of malignant and benign diagnoses can be expected. In an attempt to optimise the accuracy of CNB in this challenging setting, a large number of samples were collected, whereas the mean number of samples harvested in the previous studies varied from 1 to 4.5. Moreover, although this was apparently achieved in one previous series only (de Kerviler *et al*, 2007), fresh tissue was used for flow cytometry analysis and two cores of tissue were systematically frozen, allowing for further morphological, immunochemistry and molecular studies. Those additional samples were useful to establish a precise diagnosis in 40% of cases (data not shown).

Regarding the large differential diagnosis expected in this patient population, several end-points were considered in this study. The diagnostic yields of multisampling CNB for malignancy, lymphoma typing and complete lymphoma subtyping/grading were 100, 98 and 86%, respectively. This indicates that no case of malignancy was missed, that the diagnosis of lymphoma was established in all but one case, and a precise subtyping/grading was possible in 55/63 cases. It is important to note that the lack of precise subtyping/grading in a minority of cases did not preclude a therapeutic decision to be made for 62/63 patients (98%) with lymphoma. These rates not only compare favourably with those obtained in more homogeneous patient populations (de Kerviler *et al*, 2000, 2007; Demharter *et al*, 2001; Screaton *et al*, 2002; Balestreri *et al*, 2005; Li *et al*, 2005; Sklair-Levy *et al*, 2005; de Larrinoa *et al*, 2007; Lachar *et al*, 2007) but also with results usually obtained by complete lymph node analysis. On the other hand, the diagnostic yield of CNB for diagnosis of benignity was only 53%, confirming that a definitive diagnosis for a benign disorder is a difficult task. However, careful follow-up and/or surgical exploration allowed lymphoma to be excluded in all uncertain cases (*n*=8). This further demonstrates that CNB, performed in patients with suspected LPD in the context of a large differential diagnosis, permitted not only a precise diagnosis of lymphoma to be made but also lymphomas to be distinguished from other malignant diseases and benign processes.

CNB may have some intrinsic weakness for lymphomas with complex architecture, few or polymorphic tumour cells or a strong reactive cellular process as exemplified by the difficulty in defining the subtype of Hodgkin's lymphoma and in grading all cases of FL. The diagnostic value of CNB in certain other situations (e.g., T-cell-enriched B-cell or T-cell lymphoma) could not be established by this study, because of the non-selective accrual of patients in our institution and, as a result, the low number of such lymphoma entities. However, T-cell lymphomas tend to be rare, and even in reference centres, at least in Europe and North America, usually constitute less than 5% of lymphomas (de kerviler *et al*, 2007).

Such results also highlight the importance of a coordinated approach between radiologists, pathologists and oncologists/haematologists to optimise the diagnosis of suspected lymphomas. Moreover, such accuracy with CNB requires review all specimens by a haemato-histopathologist.

## Conclusion

In a primary care setting, multisampling large-cutting CNB is a very accurate procedure for confirming malignancy in patients with suspected lymphoma. CNB should be the first procedure proposed to those patients whose suspect lymph nodes or masses are deep seated or whose clinical condition is severely impaired and for those patients who reject a lymph node biopsy. CNB can also be appropriate for patients with peripheral lymph nodes/tumour masses because it is a readily available, few-invasive and safe procedure, that has a very high diagnostic yield for lymphoma typing and a high diagnostic yield for complete lymphoma subtyping/grading. This allows a therapeutic decision to be taken in most patients (98% in this series), avoiding expensive, time-consuming and chemotherapy-delaying surgery.

## Figures and Tables

**Table 1:** Distribution and diameter of biopsied lesions in 112 patients with suspected lymphoma

	**Initial presentation lymphoma (*n*=87)**	**Relapsed/transformed lymphoma (*n*=25)**
*Lymphoid sites*		
Retroperitoneum	23 (20–130 mm; 40 mm)	5 (15–100 mm; 48 mm)
Neck/cervical	13 (15–80 mm; 33 mm)	—
Mediastinum	11 (30–90 mm; 55 mm)	—
Axilla	8 (10–90 mm; 33 mm)	4 (20–40 mm; 29 mm)
Groin	5 (20–60 mm; 42 mm)	2 (20 mm)
Supraclavicular	4 (10–60 mm; 29 mm)	1 (30 mm)
Spleen	3 (90–120 mm; 103 mm)	—
Porta hepatic	—	1 (20 mm)
		
*Non-lymphoid sites*		
Chest wall	5 (35–100 mm; 63 mm)	2 (30, 35 mm)
Abdomen	4 (50–100 mm; 72 mm)	2 (50, 60 mm)
Muscle	3 (30–60 mm; 45 mm)	2 (35, 50 mm)
Lung	2 (35, 40 mm)	2 (25, 30 mm)
Adrenal	1 (50, 80 mm)	—
Spine	2 (30, 45 mm)	—
Liver	1 (40 mm)	3 (10–20 mm; 15 mm)
Bone	1 (100 mm)	—
Kidney	1 (30 mm)	1 (35 mm)

Numbers without parentheses correspond to number of patients. Numbers in parentheses correspond to lesion diameters, or diameter ranges with mean diameters.

Lesion size is defined by largest axis diameter.

**Table 2:** Distribution of CNB results in 112 patients with suspected lymphoma

*Lymphoma with complete subtyping/grading (54)*	
DLBCL activated type	13
DLBCL germinal centre type	8
MCL	3
Anaplastic large cell lymphoma	3
Nodular sclerosis classical HL	4
Nodular lymphocyte predominant HL	2
Mixed cellularity classical HL	2
Lymphocyte-rich classical HL	1
FL grade 3	3
FL grade 2	1
FL grade 1	1
Monomorphic PTLD (DLBCL type)	1
Precursor T-lymphoblastic lymphoma	1
Angioimmunoblastic T-cell lymphoma	1
DLBCL, follicular lymphoma in transformation	1
Castelman disease	1
High-grade lymphoma, blastoid transformation	1
Lymphoplasmocytic lymphoma	1
Burkitt lymphoma	1
Precursor B-lymphoblastic lymphoma	1
CLL in transformation (Richter syndrome)	1
CLL	1
Multiple myeloma	2
	
*Lymphoma, incomplete subtyping/grading (8)*	
Classical HL	4
DLCBL	2
FL grading not specified	1
B-cell lymphoma	1
	
*Lymphoma, incomplete typing (1)*	
HL or anaplastic large cell lymphoma	1
	
*Non-lymphoma malignant diagnoses (32)*	
Epithelial tumours	23
Non-épithélial tumours	9
	
*Benign diagnoses (8)*	
Pyogenic acute inflammation with fungus	1
Granulomatous lymphadenitis (BAAR+)	3
Necrotising granulomatous inflammation (PCR :DNA of MT)	2
Non-specified periportal inflammation	1
BOOP	1
	
*Non-malignant diagnoses. Lymphoma could not be definitively ruled out on CNB results (9)*	
Mix lymphocytic infiltrate	1
Necrotising granulomatous inflammation	2
Benign fibroadipose tissue	1
Non-necrotising non-granulomatous reactive lymphadenitis	1
Acute and chronic inflammation	1
Reactive lymphoid tissue without tumour	1
Lymphoid tissue with follicular hyperplasia	2

DLBC=diffuse large B-cell lymphoma; MCL=mantle cell lymphoma; HL=Hodgkin's lymphoma; FL=follicular lymphoma; PTLD=post-transplantation lymphoproliferative disease; CLL=chronic lymphatic leukaemia; BAAR=acid-alcohol resistant bacillus; PCR=polymerase chain reaction; DNA=deoxyribonucleic acid; MT=*Mycobacterium tuberculosis*; BOOP=bronchiolitis obliterans organised pneumonia.

**Table 3:** CNB results in 10 patients with suspected relapsed/transformed lymphoma for whom the CNB diagnosis was different from initial diagnosis

**Initial diagnoses**	**Diagnoses at CNB**
MCL	High-grade lymphoma, blastoid transformation
Grade 3 FL	DLBCL
Lymphoplasmocytic lymphoma	Grade 1 FL
Lymphocyte-rich classical HL	Nodular lymphocyte predominant HL
DLBCL germinal centre subtype	CLL in transformation (Richter syndrome)
Nodular sclerosis classical HL	Grade 2 FL
HL	Nodular lymphocyte predominant HL
BCL (HIV+)	Non-specified periportal inflammation
Nodular sclerosis classical HL	BOOP
Nodular sclerosis classical HL	Reactive lymphoid tissue with follicular hyperplasia

MCL=mantle cell lymphoma; FL=Follicular lymphoma; DLBC=diffuse large B-cell lymphoma; HL=Hodgkin's lymphoma; CLL=chronic lymphatic leukaemia; BCL=B-cell lymphoma; BOOP=bronchiolitis obliterans organised pneumonia.

**Table 4:** Diagnostic work-up of nine patients with non-malignant diagnoses at CNB for whom lymphoma could not be definitively ruled out by CNB results

**CNB results**	**Final diagnostical procedure**	**Final diagnosis**
Mix lymphocytic infiltrate	Patient lost of follow-up	Histological result of CNB could not be confirmed
Necrotising granulomatous inflammation	Cultures positive for MT	Tuberculosis
Necrotising granulomatous inflammation	Bronchoscopy Cultures positive for MT	Tuberculosis
Benign fibroadipose tissue	Spontaneous resolved process on follow-up	Benign axillary lymph node
Non-necrot.. non-granulo. react. lymphadenitis	Resolved process on clinical follow-up	Benign cervical lymph node
Acute and chronic inflammation	Subsequent surgical biopsy of the identical lymph node	Acute and necrotising lymph node inflammation
Reactive lymphoid tissue without tumour	Subsequent surgical biopsy of the identical lymph node	Fibrosis and chronic lymph node inflammation
Lymphoid tissue with follicular hyperplasia	Subsequent surgical biopsy of the identical lymph node	Reactive lymph node follicular hyperplasia
Reactive lymphoid tissue with follicular hyperplasia	Subsequent surgical biopsy of the identical lymph node	Reactive lymph node follicular hyperplasia

MT=*Mycobacterium tuberculosis*.
